# Genetic diversity of Halla horses using microsatellite markers

**DOI:** 10.1186/s40781-016-0120-6

**Published:** 2016-11-17

**Authors:** Joo-Hee Seo, Kyung-Do Park, Hak-Kyo Lee, Hong-Sik Kong

**Affiliations:** 1 Genomic Informatics Center, Hankyong National University, Anseong, 17579 Korea; 2 Department of Genomic Informatics, Graduate School of Future Convergence Technology, Hankyong National University, Anseong, 456-749 Korea; 3 ChonBuk National University, Jeonju, 54896 Korea

**Keywords:** Microsatellite marker, Halla Horse, Polymorphism, Allele frequency

## Abstract

**Background:**

Currently about 26,000 horses are breeding in Korea and 57.2% (14,776 horses) of them are breeding in Jeju island. According to the statistics published in 2010, the horses breeding in Jeju island are subdivided into Jeju horse (6.1%), Thoroughbred (18.8%) and Halla horse (75.1%). Halla horses are defined as a crossbreed between Jeju and Thoroughbred horses and are used for horse racing, horse riding and horse meat production. However, little research has been conducted on Halla horses because of the perception of crossbreed and people’s weighted interest toward Jeju horses.

**Method:**

Using 17 Microsatellite (MS) Markers recommended by International Society for Animal Genetics (ISAG), genomic DNAs were extracted from the hair roots of 3,880 Halla horses breeding in Korea and genetic diversity was identified by genotyping after PCR was performed.

**Results and conclusion:**

In average, 10.41 alleles (from 6 alleles in HTG7 to 17 alleles in ASB17) were identified after the analysis using 17 MS Markers. The mean value of H_obs_ was 0.749 with a range from 0.612(HMS1) to 0.857(ASB2). Also, it was found that H_exp_ and PIC values were lowest in HMS1 (0.607 and 0.548, respectively), and highest in LEX3(0.859 and 0.843, respectively), and the mean value of H_exp_ was 0.760 and that of PIC was 0.728. 17 MS markers used in this studies were considered as appropriate markers for the polymorphism analysis of Halla horses. The frequency for the appearance of identical individuals was 5.90 × 10^−20^ when assumed as random mating population and when assumed as half-sib and full-sib population, frequencies were 4.08 × 10^−15^ and 3.56 × 10^−8^, respectively. Based on these results, the 17 MS markers can be used adequately for the Individual Identification and Parentage Verification of Halla horses. Remarkably, allele M and Q of ASB23 marker, G of HMS2 marker, H and L of HTG6 marker, L of HTG7 marker, E of LEX3 marker were the specific alleles unique to Halla horses.

**Electronic supplementary material:**

The online version of this article (doi:10.1186/s40781-016-0120-6) contains supplementary material, which is available to authorized users.

## Background

Horses (: *Equus caballus*) were first domesticated 4000 years ago and they are one of the most important animals for human and currently, about 200 horse breeds are breeding in the world. In the past years they were used as meat animals, workhorses or transportation means, but nowadays they are used for the various purposes such as improvement of life quality and development of leisure culture.

Currently about 26,000 horses are breeding in Korea and 57.2% (14,776 horses) of them are breeding in Jeju island [14].

According to the statistics published in 2010 [10], the horses breeding in Jeju island are subdivided into Jeju horse (6.1%), Thoroughbred (18.8%) and Halla horse (75.1%). Halla horses are defined as a crossbreed between Jeju and Thoroughbred horses and are used for horse racing, horse riding and horse meat production. However, little research has been conducted on Halla horses because of the perception of crossbreed and people’s weighted interest toward Jeju horses.

This stduy was conducted to establish parentage and to certify purity of Halla horses using microsatellite markers through the scientific and systemic management and to understand the value of Halla horses as genetic resources at national level, and to obtain the basic information on the genetic discrimination from other breeds and conservation of purity and genetic improvement of Halla horses.

## Methods

### Animals and DNA extraction

For the analysis of DNAs using Microsatellite, 3,880 heads of Halla horse breeding in Korea were used. Genomic DNAs were isolated and extracted by the methods described by QuickGene DNA tissue kits (FUJIFILM, Japan) and the concentration and purity of extracted genomic DNAs were measured using ND-1000 UV-Vis Spectrophotometer (NanoDrop Technologies, USA) and used for the analysis.

### Information on Microsatellite (MS) Markers

Genetic diversity of Halla horse was identified using 17 Microsatellite (MS) Markers (AHT4, AHT5, ASB2, ASB17, ASB23, CA425, HMS1, HMS2, HMS3, HMS6, HMS7, HTG4, HTG6, HTG7, HTG10, LEX3, VHL20) recommended by International Society for Animal Genetics (ISAG).

### Composition of multiplex-PCR and PCR procedure

Multiplex-PCR was performed using Equine Genotpyes Panel 1.1 Kit (Thermo SCIENTIFIC) for genotyping of 17 MS Markers. By the manufacturer’s instructions, to the reaction mixtures Genomic DNA (1.0 ng/μl) 2 μl, Mater Mix 9 μl, and Primer Mix 9 μl were added, making a total of 20 μl and using GeneAmp PCR system 9700 (Applied Biosystems, USA) PCR was performed. PCR was performed in an initial denaturation at 98 °C for 3 min, followed by 30 cycles of 15 s at 98 °C, 75 s at 60 °C and 30s at 72 °C. The final extension step was at 72 °C for 5 min and then cooled to 4 °C.

### Genotyping of Microsatellite (MS)

Using Hi-Di™ formamide, amplified PCR products were diluted to 50 ~ 100 times, and the diluted PCR products were diluted using Hi-Di™ formamide and GeneScan™-500LIZ™ size standard. After the capillary electrophoresis was performed using Genetic Analyzer 3130xl (Applied Biosystem, USA), the size of each MS marker was determined using GeneMapper version 4.1 (Applied Biosystems, USA). Data on the determined alleles were collected individually and applied to statistical analysis using Microsoft Excel (Microsoft, USA). Also, by the international equine comparison test standards recommended by ISBC (International Stud Book Committee) and ISAG (International Society of Animal Genetics) genotyping was performed and alphabetic allele nomenclature was applied.

### Statistical analysis of data

#### Frequency, heterozygosity and polymorphism information content

Using Microsatellite Toolkit software [15] and Cervus ver 3.0 program [13], number of alleles, expected and observed heterozygosity (H_exp_ and H_obs_) and Polymorphism information content (PIC) value were calculated.

#### Frequency of identical individuals

Using API-CALC ver 1.0 program [1], expected probabilities of appearance frequency of identical individuals in random individuals (PI), random half sibs (PI_half-sibs_) and random full sibs (PI_fullsibs_) mating population were calculated.

## Results and discussion

### Polymorphism of microsatellite markers

For 3,880 heads of Halla horses breeding in Korea, the number of alleles, observed heterozygosity (H_obs_), expected heterozygosity (H_exp_) and Polymorphism Information Content (PIC) values of 17 MS markers were calculated (Table 1).

**Table 1:** No. of Allele, Heterozygosity (observed and expected) and PIC value of microsatellite markers in Halla horses

Marker	No of allele	Hobs	Hexp	PIC
AHT4	11	0.843	0.835	0.814
AHT5	10	0.810	0.808	0.781
ASB2	13	0.857	0.843	0.824
ASB17	17	0.790	0.770	0.746
ASB23	13	0.802	0.814	0.789
CA425	11	0.623	0.623	0.598
HMS1	11	0.612	0.607	0.548
HMS2	11	0.760	0.748	0.717
HMS3	8	0.746	0.789	0.760
HMS6	7	0.743	0.736	0.697
HMS7	8	0.748	0.765	0.728
HTG4	7	0.625	0.619	0.569
HTG6	11	0.728	0.711	0.659
HTG7	6	0.750	0.749	0.706
HTG10	13	0.843	0.843	0.825
LEX3	11	0.641	0.859	0.843
VHL20	9	0.815	0.803	0.778
Mean	10.41	0.749	0.760	0.728

The mean number of alleles for 17 MS markers was 10.41 with a range from 6 (HTG7) to 17(ASB17). Cho [6] reported that there were 7.35 alleles in average with a range from 5(HTG4, HTG7) to 10 (ASB17) in the analysis of genetic characteristics and Genetic Relationship for Jeju horse, Mongolian horse, Thoroughbred and Warmblood when used the same 17 markers used in this study. Similar results were also reported by Cho [7], Cho et al. [5] and Lee et al. [11].

The mean value of H_obs_ was 0.749 with a range from 0.612(HMS1) to 0.857(ASB2). Also, H_exp_ and PIC values were lowest in HMS1 (0.607 and 0.548, respectively), and highest in LEX3 (0.859 and 0.843, respectively). For CA425 the number of alleles was relatively high (11), while H_obs_, H_exp_ and PIC values were relatively low (0.623, 0.623 and 0.598, respectively).

While the number of alleles in HTG7 was lowest (6), H_obs_, H_exp_ and PIC values were higher than those of CA425 (0.750, 0.749 and 0.706, respectively), suggesting that alleles in HTG7 were evenly distributed.

Botstein et al. [3] reported that when the expected heterozygosity was above 0.6 and Polymorphism Information Content (PIC) value of MS marker was above 0.5 it is considered as markers with high diversity. Therefore, the 17 MS markers used in this studies are considered as appropriate markers for the analysis of genetic diversity of Halla horses.

### The expected probability of identity values

Using 17 MS markers (AHT4, AHT5, ASB2, ASB17, ASB23, CA425, HMS1, HMS2, HMS3, HMS6, HMS7, HTG4, HTG6, HTG7, HTG10, LEX3, VHL20), the expected probability of identity values in Halla horses were calculated (Table 2). When assumed as random mating population the frequency was 5.90 × 10^−20^ and when assumed as half sib and full-sib mating population the frequencies were 4.08 × 10^−15^ and 3.56 × 10^−8^, respectively. From these results, 17 MS markers used in this study can be applicable for Individual Identification and Parentage Verification of Halla horses.

**Table 2:** The expected probability values among genotypes of random individual (PI) for discrimination horse lines using markers

No. of Marker	Random	Half-sib	Sib
1	3.20 × 10^−2^	8.09 × 10^−2^	3.09 × 10^−1^
2	1.22 × 10^−3^	7.33 × 10^−2^	9.77 × 10^−2^
3	4.76 × 10^−5^	6.70 × 10^−2^	3.11 × 10^−2^
4	2.04 × 10^−6^	6.52 × 10^−5^	1.00 × 10^−2^
5	1.08 × 10^−7^	7.29 × 10^−6^	3.37 × 10^−3^
6	6.10 × 10^−9^	8.50 × 10^−7^	1.14 × 10^−3^
7	3.49 × 10^−10^	1.01 × 10^−7^	3.89 × 10^−4^
8	2.30 × 10^−11^	1.31 × 10^−8^	1.36 × 10^−4^
9	1.58 × 10^−12^	1.83 × 10^−9^	4.87 × 10^−5^
10	1.30 × 10^−13^	2.73 × 10^−10^	1.78 × 10^−5^
11	1.23 × 10^−14^	4.44 × 10^−11^	6.66 × 10^−6^
12	1.03 × 10^−15^	7.00 × 10^−12^	2.49 × 10^−6^
13	9.90 × 10^−17^	1.18 × 10^−12^	9.47 × 10^−7^
14	1.20× 10^−17^	2.29 × 10^−13^	3.78 × 10^−7^
15	1.79 × 10^−18^	5.66 × 10^−14^	1.69 × 10^−7^
16	3.10 × 10^−19^	1.48 × 10^−14^	7.66 × 10^−8^
17	5.90 × 10^−20^	4.08 × 10^−15^	3.56 × 10^−8^

### Allele frequency of microsatellite markers

#### AHT4

**Fig. 1:**
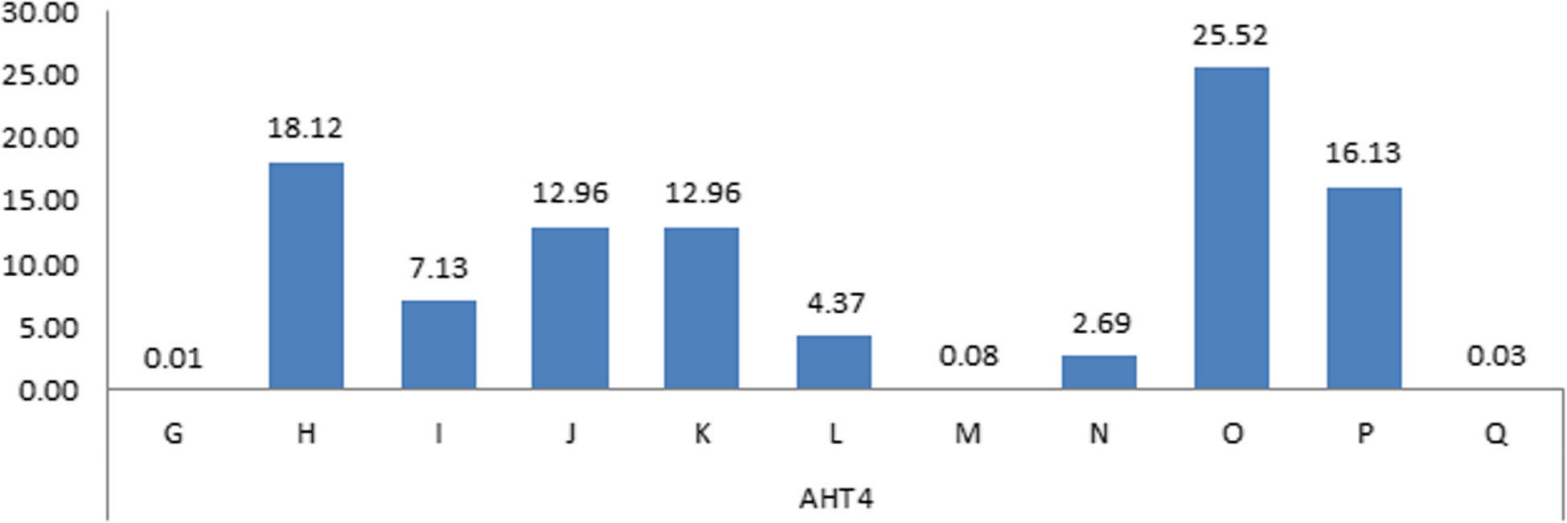
Alleles and allele’s frequencies for the Microsatllite *AHT4* in the studied breeds

In Halla horses, a total of 11 alleles in AHT4 marker were detected and their frequencies are shown in Fig. 1. Frequencies of 3 alleles (O, H and P) were relatively high (25.52, 18.12 and 16.13, respectively), while those of 3 alleles (G, Q, and M) were low (0.01, 0.03, 0.08, respectively). It was reported that repeat structure of AHT4 was (AC)_n_AT(AC)_n_ (Genbank : Y07733) and the allele with repeat of (AC)_18_AT(AC)_9_ was allele K and the frequency was similar to that of this experiment processed by alphabetic nomenclature [7, 11, 17]. Allele G was not detected when alleles of AHT4 were investigated for the 9,094 horses of 35 breeds including Thoroughbred [17]. However, Cho [7] reported that although low in allele frequency (0.0002), it was detected in the parentage test of Thoroughbred horses breeding in Korea. This result suggests that if the genetic improvement and breeding goals are established through the research on allele G in Thoroughbred and Halla horses breeding in Korea, it will be useful for the discrimination of domestic and foreign breeds of horse.

#### AHT5

**Fig. 2:**
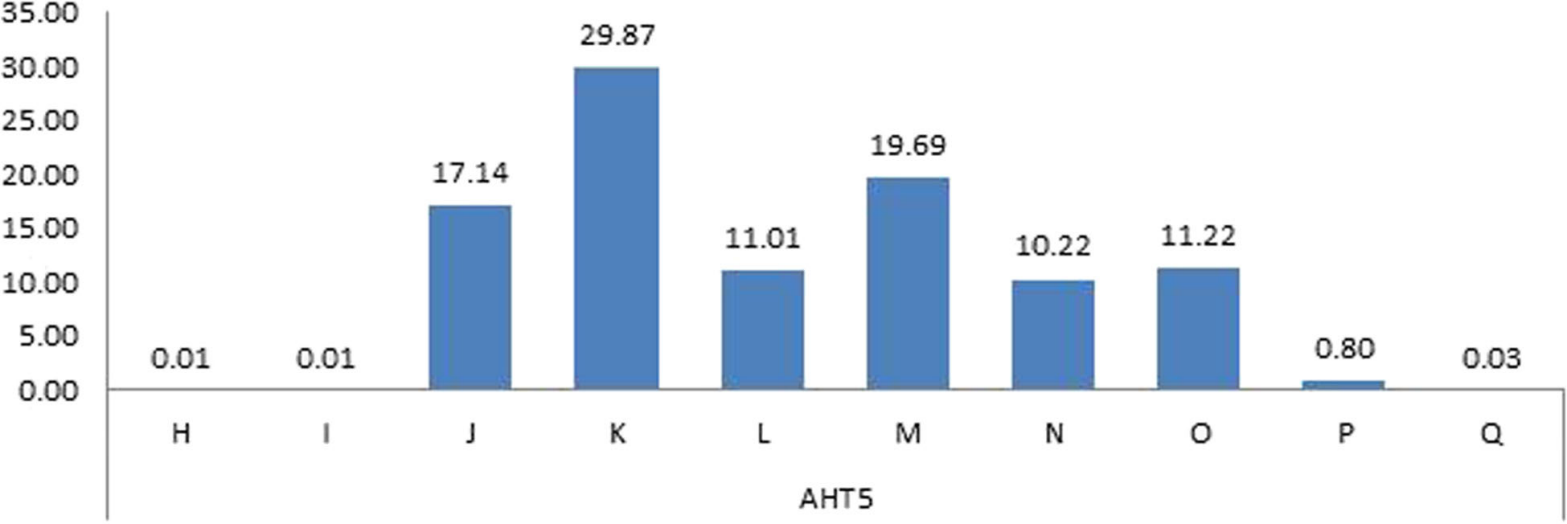
Alleles and allele’s frequencies for the Microsatllite *AHT5* in the studied breeds

AHT5 has simple dinucleotide repeats of (GT)_n_ (Genbank : Y07732)) and in Halla horses a total of 10 alleles were detected in AHT5 marker. Frequency of allele is shown in Fig. 2. The frequency of allele K was high (29.87), while frequencies of allele H and I were low (0.01). Similar results were reported in Korea, but in the studies conducted in foreign countries the frequencies of allele J and N were high (0.24 and 0.22, respectively) [17]. Also, for allele Q, it was low (0.0002) as in allele G of AHT4 in the parentage test of Thoroughbred breeding in Korea. Therefore, since allele Q of AHT5 was detected in Thoroughbred and Halla horses in Korea, further research on allele Q is needed to obtain the useful information for the discrimination of domestic and foreign horse breeds as allele G of AHT4.

#### HMS2

**Fig. 3:**
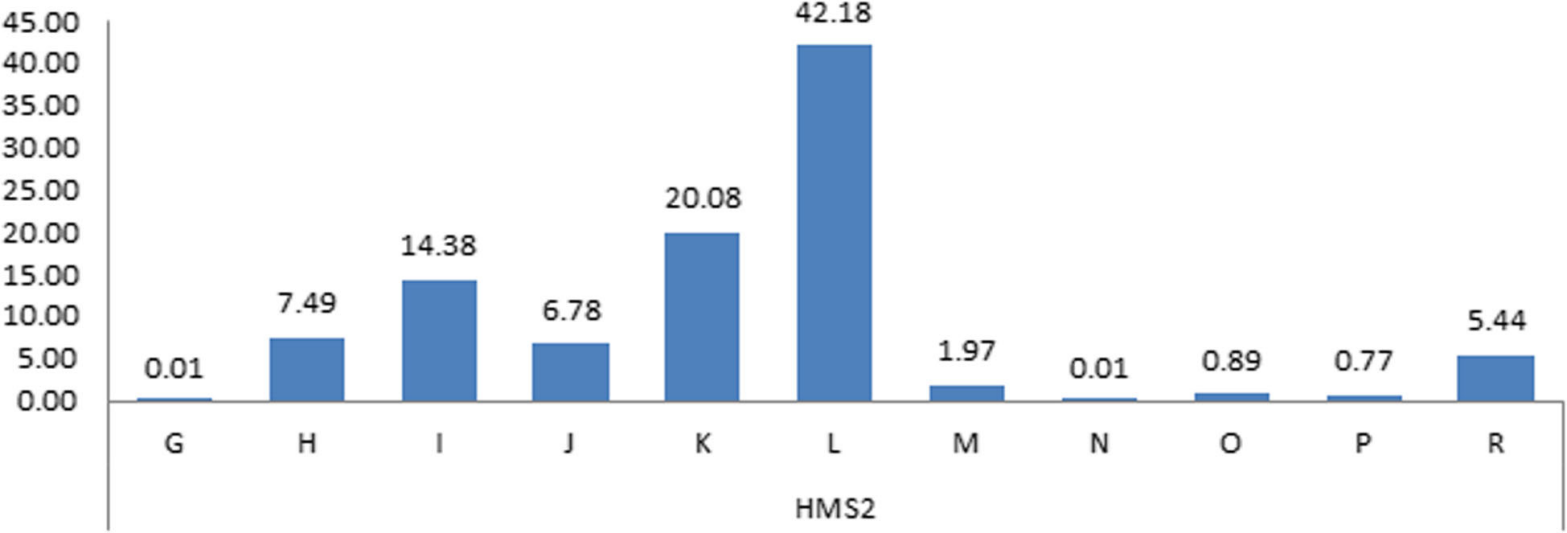
Alleles and allele’s frequencies for the Microsatllite *HMS2* in the studied breeds

HMS2 has dinucleotide repeats of (CA)_n_(TC)_2_) (Genbank : X74631)) and a total of 11 alleles were detected in HMS2 marker of Halla horses and their frequencies are shown in Fig. 3. The frequencies were high in the order of allele L, K and I (42.18, 20.08 and 14.38, respectively), while those of allele N and G were lowest (0.01). Van de Goor et al. [17] reported that in 35 horse breeds the frequencies in HMS2 were high in allele of K and H and frequencies of allele Q, S and U which were not detected in Halla horses were low. Also, although low (0.01), allele G was detected only in Halla horses. This specific allele can be used for the basic information for breed discrimination. The other markers were written into Additional file 1.

## Conclusion

Although several studies [2, 8, 9, 12] reported population studies on the genetic pedigree structure of several horse breeds, only limited information is available about data needed in casework such as the power of identity and breed assignment [4, 16]. In this study, allele M and Q of ASB23 marker, G of HMS2 marker, H and L of HTG6 marker, L of HTG7 marker, E of LEX3 marker confirmed as the specific alleles unique to Halla horses. This result is considered that utilize for the basic information on genetic resource and genetic relationship analysis of Halla horses. Also, it is thought to used as scientific evidence that prove pedigree establishment, genetic differentiation and inherency of halla horses. Finally, It is considered to be a useful data in the improved ability to breed Halla horses.

## Additional file

Additional file 1: Allele frequency of Microsatellite Markers. (DOCX 128 kb)
