# Supplementary material for: Genetic diversity of Halla horses using microsatellite markers
Source: J Anim Sci Technol. 2016 Nov 17;58:40. doi: 10.1186/s40781-016-0120-6 (PMC5114825; doi:10.1186/s40781-016-0120-6)
Supplement: Additional file 1: — Allele frequency of Microsatellite Markers. (DOCX 128 kb) [file 40781_2016_120_MOESM1_ESM.docx]

1. **Allele frequency of Microsatellite Markers**
2. ***(1) ASB2***
3. ASB2 has simple dinucleotide repeats of ((GT)_n_)(Genbank : X93516)) and a total of 13 alleles were detected in ASB2 marker of Halla horses and their frequencies are shown in Figure 3. The frequencies were high in the order of allele N, Q and K (22.09, 18.54 and 16.88, respectively), while the frequency of allele J was lowest (0.09).
4.
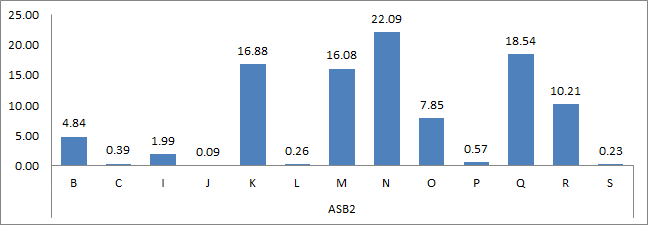

5. Figure 1. Alleles and their frequencies for the Microsatellite *ASB2* in Halla horses.
6. ***(2) ASB17***
7. ASB17 has simple dinucleotide repeats of ((AC)_n_)(Genbank : X93531)) and a total of 17 alleles were detected in ASB17 marker of Halla horses and their frequencies are shown in Figure 4. The frequencies were high in the order of allele N, R and O (40.62, 15.64 and 14.14, respectively), while those of allele T, U and V were lowest (0.01).
8.
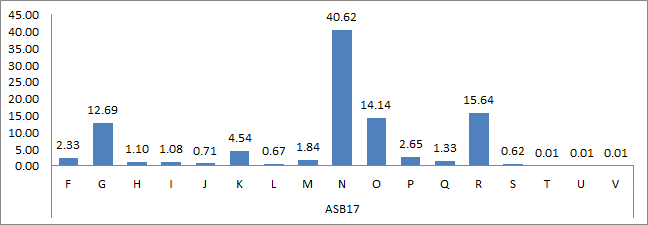

9. Figure 2. Alleles and their frequencies for the Microsatellite ASB17 in Halla horses.
10. ***(3) ASB23***
11. ASB23 has simple dinucleotide repeats of ((TG)_n_)(Genbank : Y93537)) or compound repeats in which TT is inserted between base pairs ((TG)_n_TT(TG)_4_)(Genbank : NW_001799714)) and a total of 13 alleles were detected in ASB23 marker of Halla horses and their frequencies are shown in Figure 5. The frequencies were high in the order of allele K, J and S (26.22, 25.39 and 14.97, respectively), while low in the order of allele O, H and V (0.01, 0.03 and 0.05, respectively). Although the frequencies of allele M and O in Halla horses were low, they were not detected in other breeds. Therefore, allele M and O of ASB23 marker seem be the specific alleles for Halla horses and can be used as useful information.
12.
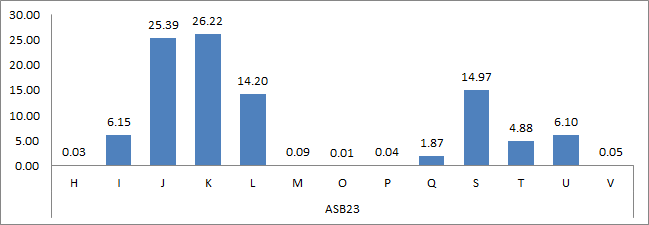

13. Figure 3. Alleles and their frequencies for the Microsatellite *ASB23* in Halla horses.
14. ***(4) CA425***
15. CA425 has simple dinucleotide repeats of ((GT)_n_)(Genbank : U67406)) and a total of 11 alleles were detected in CA425 marker of Halla horses and their frequencies are shown in Figure 6. The frequency of allele N was fairly high (58.43) compared with those of other alleles.
16.
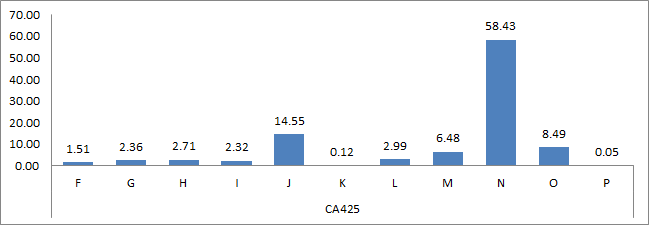

17. Figure 4. Alleles and their frequencies for the Microsatellite *CA425* in Halla horses.
18. ***(5) HMS1***
19. HMS1 has simple dinucleotide repeats of ((TG)_n_)(Genbank : X74630)) and a total of 11 alleles were detected in HMS1 marker of Halla horses and their frequencies are shown in Figure 7. The frequency of allele M was fairly high (54.63) compared with those of other alleles.
20.
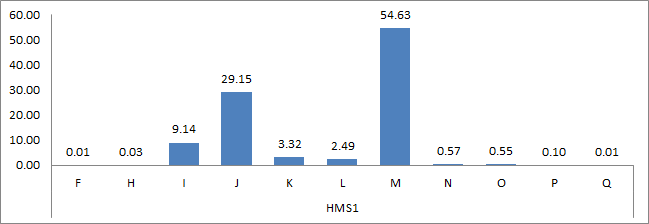

21. Figure 5. Alleles and their frequencies for the Microsatellite HMS1 in Halla horses.
22. ***(6) HMS3***
23. Dinucleotide repeats of HMS3 are (TG)_2_(CA)_2_TC(CA)_n_ and (TG)_2_(CA)_2_TC(CA)_n_GA(CA)_5_ (Genbank : X74632) and a total of 11 alleles were detected in HMS3 marker of Halla horses and their frequencies are shown in Figure 9. The frequencies were high in the order of allele I, P and M ( 32.23, 24.56 and 15.15 respectively), while lowest in allele S( 0.08)
24.
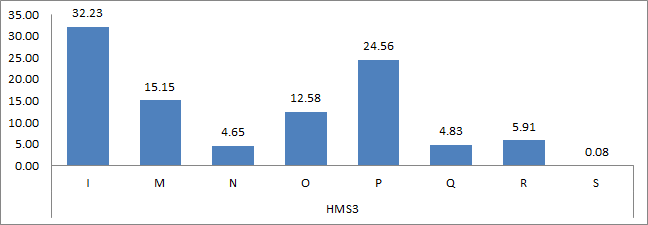

25. Figure 6. Alleles and their frequencies for the Microsatellite HMS3 in Halla horses.
26. ***(7) HMS6***
27. HMS6 has simple dinucleotide repeats of ((GT)_n_)(Genbank : X74635)) and a total of 7 alleles were detected in HMS6 marker of Halla horses and their frequencies are shown in Figure 10. The frequency of allele P was fairly high (39.72), while that of allele Q was lowest in Halla horse (0.01). The frequency of allele L was low in Halla horses, but it was relatively high (0.21) in a proposal for standardization in forensic equine DNA typing (Van de Goor et al., 2009). Raising environment and genetic flows seem to be the reasons for that.
28.
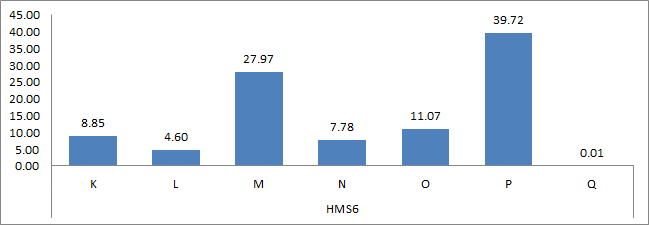

29. Figure 7. Alleles and their frequencies for the Microsatellite HMS6 in Halla horses.
30. ***(8) HMS7***
31. HMS7 has dinucleotide repeats of (AC)_2_(AC)_n_(Genbank : X74636) and a total of 8 alleles were detected in HMS7 marker of Halla horses and their frequencies are shown in Figure 11. The frequencies were high in the order of allele L (33.52), O(25.72), M(16.56) and N(15.57), while lowest in allele P ( 0.32).
32.
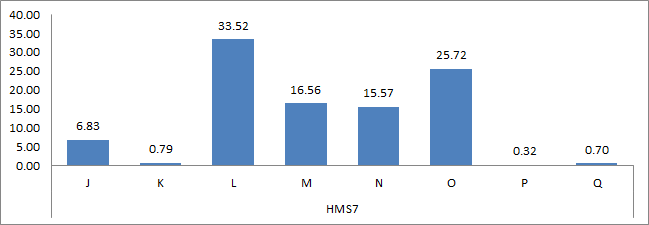

33. Figure 8. Alleles and their frequencies for the Microsatellite HMS7 in Halla horses.
34. ***(9) HTG4***
35. The repeats of HTG4 are (TG)_n_AT(AG)_5_AAG(GA)_5_ACAG(AGGG)_3_(Genbank : AF169165) and a total of 8 alleles were detected in HTG4 marker of Halla horses and their frequencies are shown in Figure 12. The frequency of allele M was relatively high (54.79) compared with that of other breeds and the frequencies were high in the order of allele K (26.70) and L (7.82).
36.
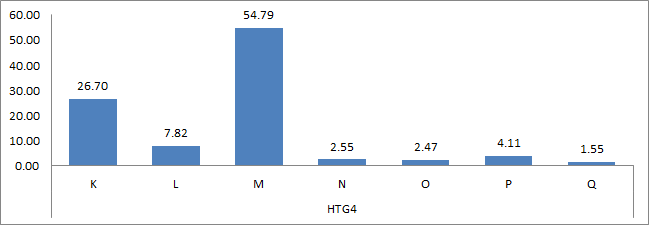

37. Figure 9. Alleles and their frequencies for the Microsatellite HTG4 in Halla horses.
38. ***(10) HTG6***
39. HTG6 has simple dinucleotide repeats of ((TG)_n_)(Genbank : AF169167)) and a total of 11 alleles were detected in HTG6 marker of Halla horses and their frequencies are shown in Figure 13. The frequencies of alleles were high in the order of allele J(36.26), O(32.26) and G(22.50) and those of others were fairly low (from 0.01 to 4.07). Frequencies of allele H and L were 0.03 and 0.01, respectively and they were detected only in Halla horses.
40.
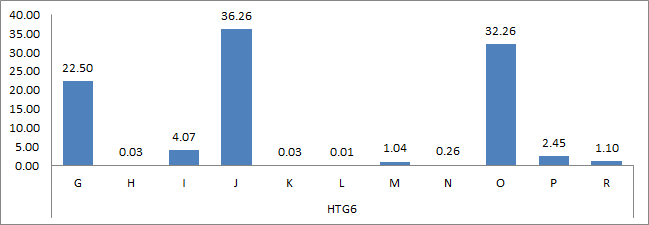

41. Figure 10. Alleles and their frequencies for the Microsatellite HTG6 in Halla horses.
42. ***(11) HTG7***
43. HTG7 has simple dinucleotide repeats of ((GT)_n_)(Genbank : AF169291)) and a total of 6 alleles were detected in HTG7 marker of Halla horses and their frequencies are shown in Figure 14. The frequencies of alleles were high in the order of allele O (32.69), K(25.97) and N(24.63) and lowest in allele L( 0.04). No frequency on allele L was reported in other research. Therefore, this result on the breed- specific allele can be used for the basic information for breed discrimination.
44.
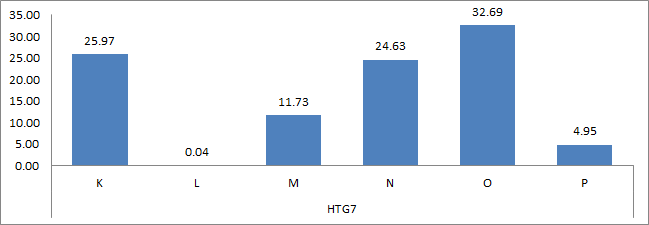

45. Figure 11. Alleles and their frequencies for the Microsatellite HTG7 in Halla horses.
46. ***(12) HTG10***
47. HTG10 has simple dinucleotide repeats of ((TG)_n_) and compound repeats of (TATC(TG)_n_)(Genbank : AF169294) and a total of 12 alleles were detected in HTG 10 marker of Halla horses and their frequencies are shown in Figure 15. The frequencies of alleles were high in the order of allele O (25.50), I(17.08) and L(15.12) and low in the allele J (0.21) and T( 0.25).
48.
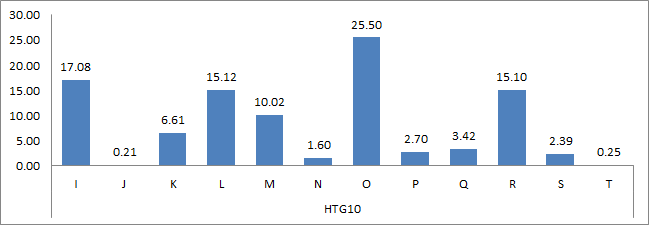

49. Figure 12. Alleles and their frequencies for the Microsatellite HTG10 in Halla horses.
50. ***(13) LEX3***
51. LEX3 has simple dinucleotide repeats of (TG)_n_)(Genbank : AF075607) and a total of 11 alleles were detected in LEX3 marker of Halla horses and their frequencies are shown in Figure 16. For LEX3, wide range of alleles were detected and the frequencies of alleles of G (0.04), E(0.06) and K(1.52) were low. In a proposal for standardization in forensic equine DNA typing (Van de Goor et al., 2009), allele Q was detected which was not detected in Halla horse, while allele E was not detected which was detected in this experiment. Therefore, allele E can be used as a useful allele for the discrimination of horse breeds.
52.
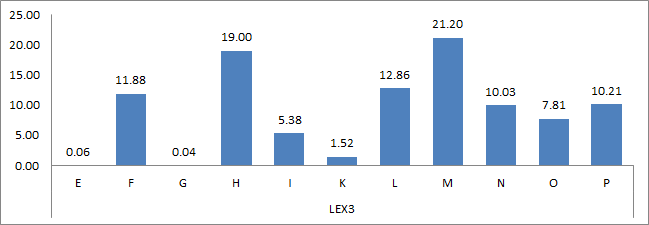

53. Figure 13. Alleles and their frequencies for the Microsatellite LEX3 in Halla horses.
54. ***(14) VHL20***
55. VHL20 has simple dinucleotide repeats of ((TG)_n_)(Genbank : X75970) and a total of 9 alleles were detected in VHL20 marker of Halla horses and their frequencies are shown in Figure 17. The frequencies of alleles were high in the order of allele I (29.06) and M (27.60) and low in allele J, Q and O (1.86, 2.59 and 3.09, respectively).
56.
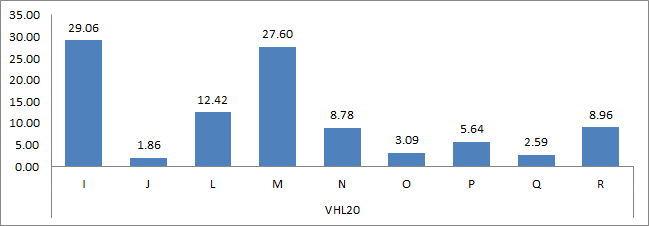

57. Figure 14. Alleles and their frequencies for the Microsatellite VHL20 in Halla horses.
